# Evolutionary toxicology in an omics world

**DOI:** 10.1111/eva.12462

**Published:** 2017-02-20

**Authors:** Elias M. Oziolor, John W. Bickham, Cole W. Matson

**Affiliations:** ^1^ Department of Environmental Science Center for Reservoir and Aquatic Systems Research (CRASR), and the Institute for Biomedical Studies Baylor University Waco TX USA; ^2^ Department of Wildlife and Fisheries Science Texas A&M University College Station TX USA

**Keywords:** eDNA, evolution, genomics, phenotypic plasticity, toxicology, transcriptomics

## Abstract

Evolutionary toxicology is a young field that has grown rapidly in the past two decades. The potential of this field comes from the ability to link chemical contamination to multigenerational and population‐wide effects in various species. The advancements and rapidly decreasing costs of ‐omic tools are improving the power and resolution of evolutionary toxicology studies. In this manuscript, we aim to address the trajectories and perspectives for conducting evolutionary toxicology studies with ‐omic approaches. We discuss the complementarity of using multiple ‐omic tools (genomics, eDNA, transcriptomics, proteomics, and metabolomics) for utility in understanding the toxicological relevance of adaptive responses in populations. In addition, we discuss phenotypic plasticity and its relevance to transcriptomic studies in toxicology. As evolutionary toxicology grows and expands its capacity to link toxicology with population‐wide end points, we emphasize the applications of such studies in answering questions about ecological and population health, as well as future applicability to regulation. Thus, we aim to emphasize the enormous potential for evolutionary toxicology in an ‐omics world and give perspectives on the directions of future investigations.

## 1 INTRODUCTION

The field of evolutionary toxicology is on the verge of having the incredible capability of directly linking chemical contamination in natural environments to the genetic basis for population‐wide outcomes of multigenerational exposures to contaminants. With recent improvements and new developments in the fields of genomics, transcriptomics, environmental chemistry, and mixture toxicity, evolutionary toxicology brings together these fields toward a complex understanding of contaminant effects on populations. The resulting knowledge of the trans and multigenerational outcomes of complex mixtures of contaminants on populations has tremendous potential for informing regulation and environmental monitoring. At this crucial time, this manuscript aims to provide direction and perspectives for upcoming genomic and transcriptomic studies on natural populations. Here we address the capabilities and limitations of ‐omic investigations of evolutionary toxicology, as well as to resolve terminology discrepancies that have arisen between evolutionary and toxicological sciences.

Evolutionary toxicology aims to characterize the effects of toxicants on populations using analyses that have their roots in the principles of population genetics (Bickham, 2011). This field arose from the realization that chronic exposure to anthropogenically contaminated environments over multiple generations had caused population‐wide genetic alterations in resident biota (Bickham & Smolen, 1994). As the levels of contamination in various environments continued to increase, studies of evolutionary toxicology began to focus on the outcomes of toxicants as a selective pressure in contaminated environments (Meyer, Nacci, & Di Giulio, 2002; Meyer, Wassenberg, Karchner, Hahn, & Di Giulio, 2003b; Nacci, Champlin, & Jayaraman, 2010; Noyes et al., 2009; Oziolor & Matson, 2015; Wirgin et al., 2011). In addition, technological improvements and lower costs have dramatically increased our ability to generate genomic, transcriptomic, proteomic, and metabolomics data, and facilitated studies focused on quantitating the relationship between contaminated environments as a selective stressor and changes in the genotypes of resident organisms (Cotter et al., 2016; Xie & Klerks, 2003). The first studies of genetic and physiological adaptation set the stage and principles, which guide evolutionary toxicological studies, while current technological advances aim to build and expand these methods to accommodate the large datasets now available (Bickham, 2011).

## 2 PRINCIPLES OF EVOLUTIONARY TOXICOLOGY

The four cornerstones of evolutionary toxicology were introduced by Bickham (2011) and have since served as guidelines to inform experimental design and to explain differences among populations, driven to diverge by anthropogenic contaminants. Effects such as (i) genome‐wide changes in diversity, (ii) changes in allelic or genotypic frequencies due to contaminant‐mediated selective pressure, (iii) changes in gene flow between populations, and (iv) increased mutation rates (Bickham, 2011) have been used to describe and search for patterns of differentiation. Studies in this field explore a wide range of selective pressures: metals (Xie & Klerks, 2003), polycyclic aromatic hydrocarbons (Mulvey, Newman, Vogelbein, & Unger, 2002; Ownby et al., 2002), polychlorinated biphenyls (Nacci et al., 1999; Oziolor, Bigorgne, Aguilar, Usenko, & Matson, 2014), dioxins and furans (Yuan, Courtenay, Chambers, & Wirgin, 2006), and in addition a variety of organisms (Oziolor, De Schamphelaere, & Matson, 2016a), but mostly focused on aquatic species. Previous reviews in the field focus on evolutionary research in fish (Wirgin & Waldman, 2004), across stressors (Oziolor & Matson, 2015), and the push toward quantitative genetics to link these processes to heritability and fitness of populations (Klerks, Xie, & Levinton, 2011). Given the difficulty of many of the approaches taken in evolutionary toxicology to map adaptation in populations to specific genes, genomic, and transcriptomic approaches are beginning to be more frequently employed to understand both the genetic basis and full effects of adaptations to contamination.

## 3 BRIEF HISTORY OF EVOLUTIONARY TOXICOLOGY

A relatively young field, evolutionary toxicology originates as an extension of wildlife toxicology and studies multigenerational effects of contaminants on populations (Bickham & Smolen, 1994). Through time, studies in this field have encompassed the multifaceted nature of population‐wide effects, not always in a hierarchical order. Of initial interest were the effects of contaminants on the genetics of adapted or contaminated populations compared to reference sites. These studies focused on the genetic structure of exposed aquatic populations (Theodorakis & Shugart, 1997), identification of genetic markers associated with possible increased fitness in contaminated environments (Theodorakis & Bickham, 2004), studying genetic structure in adapted fish populations (Mulvey, Newman, Vogelbein, Unger, & Ownby, 2003; Mulvey et al., 2002), as well as amphibians (Matson et al., 2006) and even small mammals (Matson, Rodgers, Chesser, & Baker, 2000) exposed to anthropogenic contamination. Complementary to those, other investigations have focused on the downstream effects on population health and functional differences in adapted populations. These studies have largely focused on aquatic populations, reporting decreased life span in adapted Atlantic tomcod (Dey, Peck, Smith, & Kreamer, 1993), liver neoplasia in adapted killifish (Vogelbein, Fournie, Van Veld, & Huggett, 1990), altered ability to properly develop and induce molecular responses to early developmental exposure in adapted Atlantic (Elskus, Monosson, McElroy, Stegeman, & Woltering, 1999; Nacci et al., 1999) and Gulf (Oziolor et al., 2014) killifish. Quantitative genetic efforts stemming largely from experimental evolutionary toxicology aim to quantify heritability and costs of adaptation to contaminants (Klerks et al., 2011; Xie & Klerks, 2003; Xie & Klerks, 2004). Some studies go further in an effort to understand the differential toxicology of multiple compounds on adapted populations. These studies have begun to unravel some of the many affected mechanisms during the evolution of resistance such as susceptibility to neoplasia (Wills et al., 2010), genotoxicity (Jung, Cho, Collins, Swenberg, & Di Giulio, 2009; Jung et al., 2011), estrogenicity (Dong, Wang, Thornton, Scheffler, & Willett, 2008; Grans et al., 2015), and oxidative stress (Harbeitner, Hahn, & Timme‐Laragy, 2013; Meyer, Smith, Winston, & Di Giulio, 2003a). Many of these pathways and systems were pursued because of mechanistic linkages among toxins, or differentially expressed or functioning pathways that explained resistance. Such a bottom‐up approach to evolutionary toxicology has proven extremely fruitful in identifying differences between adapted and reference populations, in terms of both their responses to toxins and their general physiology. With the increase in ability to perform larger ‐omic experiments in both model and nonmodel organisms (Schlotterer, Tobler, Kofler, & Nolte, 2014), the ability to attack similar questions with a top‐down genomic approach is becoming a reality.

In this manuscript, we aim to provide insight into the possibilities for ‐omic approaches to address issues in evolutionary toxicology. Here we will give a summary and commentary on the ability of genomics to elucidate the effects of adaptation in natural populations. In addition, we discuss the advantages and place of other methods such as eDNA, transcriptomics, proteomics, and metabolomics in this field. We discuss the concept of phenotypic plasticity in toxicology and possible evolutionary and nonevolutionary effects of toxins on molecular phenotypic plasticity. And lastly, we discuss the potential for evolutionary toxicology to inform the regulation of contaminants.

## 4 EVOLUTIONARY AND POPULATION GENOMICS

As mentioned above, identifying the mechanisms of adaptation to contamination can be accomplished through two different approaches: bottom‐up and top‐down. In bottom‐up studies, the specific mechanism of action of the contaminants that act as selective pressures become the target for exploration. One of the best‐known examples of this is the case of a six nucleotide deletion in the aryl hydrocarbon receptor (AhR) of Atlantic tomcod, which renders the molecule less capable of binding the toxin of concern and thus protects from the chronic toxicity in adapted populations (Wirgin et al., 2011). Mechanistic and targeted approaches in the field are extremely valuable in understanding pathways that selection could act upon or alter (Hahn, 2011). On the other hand, such approaches are less capable of identifying causation for adaptation in cases where more complex mechanisms or genetic interactions are present (Reitzel et al., 2014). In fact, while the strong mechanistic explanation in Atlantic tomcod was sufficient to explain the differential resistance, there has been no further search to find any other genes under selection in these populations (Wirgin et al., 2011). In such cases, genomic methods may be employed to conduct a top‐down approach to identify differences between genomes of reference and resistant populations without a priori knowledge of the mechanisms involved or the genomic regions impacted (Nacci, Proestou, Champlin, Martinson, & Waits, 2016).

A recent thorough review of evolutionary genomics in toxicology showcases the high power that this approach has demonstrated in identifying the genetic basis of adaptation regardless of genetic complexity (Whitehead, 2014). One aspect of genomic investigations, the quantitative trait loci (QTL) approach, links resistant phenotypes with specific genomic regions or number of loci based on crosses of resistant and reference individuals (Martinez & Levinton, 1996). This approach has been recently combined with the use of microsatellite and single nucleotide polymorphism (SNP) data and was successful in both identifying genetic regions likely under selection, as well as the gene–gene epistatic interactions that were likely to have conferred resistance in individuals (Nacci et al., 2016). In addition, combining this method with linkage disequilibrium between explored loci allows for the creation of a physical map of the explored genome and for synteny analysis between related species (Waits et al., 2016). The strengths of the QTL approach lie in the power of finding individual genome region contributions to resistance, as well as the contribution of region–region interactions to level of resistant phenotype (Nacci et al., 2016). On the other hand, while the strong roots of QTL analysis in quantitative genetics allow it to link a region of the genome to a phenotype of resistance in a quantitative fashion, it may be unable to ascribe observed resistance to specific genes (Nacci et al., 2016). As there are often multiple genes on a single QTL identified to be related to adaptation, inferences can be made as to which genes on that QTL would best explain the adaptation mechanistically, but the resolution of microsatellite and SNP data is insufficient to assign the resistance to a specific genotype. In addition, a QTL approach provides limited understanding of complex adaptations that involve multiple regions and multiple selective stressors. Thus, QTL analysis in genomics is an integral tool for assessing adaptation, in which targeted mechanistic approaches have failed to pinpoint genes under selection that explain a large portion of adaptive phenotype. While the resolution of a QTL analysis is sometimes an issue, this is also true for the complex population genomic resequencing methods discussed below. In addition, the QTL approach can be better at describing region–region interactions that form epistatic or polygenic contributions to resistance than some of the full‐genome resequencing methods described below.

Another approach of recent utility to understand selection is through population genomics. There are several ways to conduct population genomic comparisons, and while amplified fragment length polymorphism (AFLP), microsatellite, and SNP analyses are still viable methods for describing population demography, here we will focus on two more powerful approaches to study evolutionary toxicology: restriction site‐associated DNA sequencing (RADseq) and full‐genome population resequencing. RADseq, the more economical of the two methods, allows for amplification of DNA fragments surrounding restriction enzyme lysis sites on the genome. A recent review (Andrews, Good, Miller, Luikart, & Hohenlohe, 2016) outlines the best practices, as well as pros and cons of this method for population genomic studies. A RADseq approach is very strong for analyzing population demography and structure with high confidence and resolution (Andrews et al., 2016). These parameters are important for understanding the strength and impact of selective pressure in evolutionary toxicology and can help assess the population health of impacted groups. On the other hand, RADseq suffers from the limitation of only representing a small, and relatively randomly selected, portion of the genome (Lowry et al., 2016). Similar to QTLs, this approach has limited ability to identify the exact genes and rarely the actual sequences responsible for adaptation. Despite the fact that RADseq can be a good tool to study demography of populations affected by contamination, there are differing opinions on its utility in selection studies. Some groups suggest that the low genomic representation of this method make it difficult to capture and describe regions under selection (Lowry et al., 2016; Luikart, England, Tallmon, Jordan, & Taberlet, 2003). On the other hand, that view has been heavily criticized, as the necessary representation of genomes can be achieved through RADseq, given accurate experimental design (McKinney, Larson, Seeb, & Seeb, 2016). In addition, RADseq has been successfully used to study genetic regions under selection in multiple species, with a recent example in North Atlantic eels in the genus *Anguilla* (Laporte et al., 2016). Thus, we argue that RADseq has a place in evolutionary toxicology with appropriate experimental design and analysis.

A better approach for evolutionary toxicological studies than RADseq is full‐genome population resequencing. This costly method entails sequencing the full genome from multiple individuals from a population to establish nucleotide frequencies for each locus of the population's genome and compare those between impacted and reference populations. While the cost of this method is higher than RADseq, whole genome resequencing is becoming more affordable at an increasing rate, as the cost of sequencing a genome has decreased 10,000 fold in the past 15 years. Population resequencing allows for full comparisons between populations at each nucleotide, which gives it enormous power for analyzing population structure, demography, divergence, and identifying specific sequences associated with resistant phenotypes. Such projects are currently underway in both *Fundulus heteroclitus* (Reid et al., 2016) and *F. grandis*, and represent one of the most in‐depth approaches in evolutionary toxicology. The power of this approach is exemplified in a recent study of aging in turquoise killifish, *Nothobranchius furzeri*, which identified the specific genes responsible for an incredibly rapid generation turnover in this fish (Valenzano et al., 2015). Studies suggest that in order to be able to predict many population‐wide statistics with power, at least 20–30 individuals per population need to be sequenced in order to reliably measure heterozygosity and to test for Hardy–Weinberg equilibrium at all loci (Luikart et al., 2003). On the other hand, the specific genetic changes of very recent selective sweeps are unavoidably difficult to pinpoint even with full‐genome population resequencing (Reid et al., 2016). Such sweeps result in portions of the genome to appear to be under selection because of genetic hitchhiking surrounding the causative region. Thus, even with large sample size and coverage, the specific genes, rather than regions under selection, will prove difficult to find and are only obvious in rare cases (Reid et al., 2016).

An alternative method of full‐genome population resequencing, which can allow for evolutionary genomic studies to still find genotypes under selection, but at a low cost is Pool‐seq (Schlotterer et al., 2014). In this method, instead of sequencing multiple individuals from a population separately from each other, a pool of individuals is sequenced as a single sample from which allele frequencies at a locus can be estimated (Schlotterer et al., 2014). This approach works best for estimating single SNP sequences in large pools (>40 individuals, with 100 recommended) and for higher genome coverage (20×; Schlotterer et al., 2014). While this method is much more economical than full‐genome population resequencing, it offers similar resolution and strengths. The drawbacks stem from the inability of the method to map variable SNPs back to individuals in the populations examined. While that is a benefit of full‐genome population resequencing, it is not always necessary when examining large genetic sweeps in populations. Pool‐seq has been successfully applied in selection studies and the description of transposable element migration in several *Drosophila* species (Boitard, Schlotterer, Nolte, Pandey, & Futschik, 2012; Fabian et al., 2012; Kofler, Betancourt, & Schlotterer, 2012; Orozco‐TerWengel et al., 2012). This method may allow evolutionary toxicology genomic comparisons to be performed more economically.

Some difficulties associated with both RADseq and even more so with population resequencing are the computational challenges of building a reliable representation of the genome. A quality reference genome is integral to population resequencing studies and reduces the computational difficulty in aligning reads. As evolutionary toxicology studies are often performed with nonmodel organisms, the cost of building a reference genome in addition to sequencing many individuals from multiple populations can become overwhelming. In addition, the assembly of a reference genome requires specialized bioinformatics support and access to a computing cluster. On the other hand, recent innovations in genome sequencing technologies show promise in alleviating the computational difficulties associated with reference genome building. Pacific Biosciences and Oxford Nanopore Technologies are currently offering technologies that can sequence up to 15 kilobases (kb) and >100 kb DNA fragments, respectively. Lower costs and longer read lengths will allow for a much less computationally challenging and less expensive reference genomes for nonmodel organisms. While these technologies allow for faster and more reliable genome building, gene annotations and gene model building in nonmodel organisms remains a difficult task in reference genome assembly.

There is an immense applicability for population evolutionary genomics in evolutionary toxicology in both experimental evolution and evolution in the natural environment. A detailed review of evolutionary processes in response to variable stressors as studied with multigenerational genomic population resequencing can be seen in Barrick and Lenski (2013). In addition to experimental evolution, some studies found specific mutated genes and regions under selection (provided large enough coverage of sequencing) in wild populations with variable resistance due to adaptation to contamination (Reid et al., 2016). While these studies are not capable of inferring gene–gene interactions that may contribute to resistance as well as QTL studies, they have the ability to elucidate the variability in sequences that lead to differential sensitivity to contaminants, including their fixation in populations (Reid et al., 2016). In addition, such studies can fully explore the effects of degraded habitats on the complete genome and possibly understand further fitness costs and cross‐resistances of adapted populations through this approach.

Population genomic studies, in conjunction with quantitative genetic experiments, such as QTL analysis, will significantly increase our potential to completely understand the genetics of evolutionary toxicology. In addition to these advantages, population genomics can also inform for population adaptation potential, utilizing standing genetic variation to predict the sensitivity of species or populations to certain chemicals. Linking sequences and future population‐wide responses to pollution is one of the ultimate goals of toxicology, and population genomics is a step in that direction.

## 5 ENVIRONMENTAL DNA

Our understanding of the population‐wide effects of contaminants in an environment very often hinges on thorough explorations of one species that has adapted to the contamination present (Nacci et al., 1999; Oziolor et al., 2014; Yuan et al., 2006). The full impact of a contaminated environment can be missed when not exploring multiple species; however, few studies utilize multiple species (Brammell, Price, Birge, & Elskus, 2004). One future possibility to understand the genetic impacts of contaminated environments on populations of many species is through the use of environmental DNA (eDNA).

eDNA represents a novel method of sampling genetic information from resident biota that has not yet been applied in studies of evolutionary toxicology. Using this method, genetic material is obtained directly from environmental samples such as water or sediment. The sources of DNA include the shed cells from skin, feces, urine, saliva, and feathers or decomposing tissues of dead organisms, as well as microscopic organisms like plankton (Foote, Hofreiter, & Morin, 2012a; Matsui, Honjo, & Kawabata, 2001). Obtaining sequence data from eDNA has been facilitated by the use of next‐generation sequencing (NGS) technologies and DNA barcode databases (Cowart et al., 2015; Ward, 2012). Metabarcode analyses, which focus primarily on biodiversity assessments, can be complemented by methods such as quantitative PCR (qPCR) assays and SNP analyses, which focus on a particular species and known variable nucleotide positions (Foote et al., 2012a; Jerde, Mahon, Chadderton, & Lodge, 2011).

Studies of eDNA have mainly focused on questions of species diversity in freshwater systems (Hajibabaei, Shokralla, Zhou, Singer, & Baird, 2011; Shaw et al., 2016), and to a lesser degree in marine systems including, for example, fish (Thomsen et al., 2012) and marine mammals (Foote et al., 2012b). A number of studies have developed lists of species detected within ecosystems or communities, but there are other potential applications of these methods. In a study to detect fish in a 4.5‐million‐liter aquarium at the Monterey Bay Aquarium, investigators accurately identified all bony fish species (Kelly, Port, Yamahara, & Crowder, 2014) and showed that the relative abundances of eDNA sequences were statistically correlated with the rank order of species' biomass. Further refinements of these methods will likely lead to more accurate abundance estimates, and both species diversity and abundance could be used to show the effects of contaminant exposure in studies of ecotoxicology. Because eDNA studies provide nucleotide sequence data similar to pooled samples of organisms, addressing issues related to evolutionary toxicology is a matter of experimental design, including the selection of appropriate sentinel species, genes, and populations to be sampled with different exposure histories (Shaw et al., 2016). These issues have been discussed more fully in previous papers such as Bickham (2011). What is new here is the method of sampling and the research avenues it opens to the evolutionary toxicologist. Because eDNA potentially samples all of the species in a community, there are wide options for determining appropriate sentinel species including rare and endangered ones, common species that might be difficult to collect, and microscopic and unicellular species. In many cases, these would not be readily available for genetic analyses using conventional collecting methods. In other cases, such as in extreme environments, obtaining samples might be too expensive or dangerous. Eventually robots like autonomous underwater vehicles might be used to obtain water or sediment samples from extreme environments.

There are limitations to eDNA studies as well. Presently, only short DNA segments can be analyzed because of the complex milieu of DNA that is extracted from environmental samples. This might change in the future, but for now this limits the genes that can be analyzed to ones that are highly variable. As such, most eDNA studies focus on mtDNA, both because it is highly variable and because there are large public databases of mtDNA gene sequences.

## 6 OTHER ‐OMICS AND EVOLUTIONARY TOXICOLOGY

Transcriptomics provides insight into molecular mechanisms that underlie responses and toxicity and can be one of the ways that ecotoxicology aims to understand exposures and their effects on individuals and ultimately populations (Schirmer, Fischer, Madureira, & Pillai, 2010). On the other hand, genetic variation can alter the response of toxin activated genes and pathways (Bozinovic, Sit, Di Giulio, Wills, & Oleksiak, 2013; Reid et al., 2016; Whitehead, Pilcher, Champlin, & Nacci, 2012; Whitehead, Triant, Champlin, & Nacci, 2010). Thus, it becomes imperative to understand the genetic variation within a population and how that affects responsiveness to a specific toxin. Changes in the frequency of toxin‐responsive alleles with differential sensitivities can alter the overall population responsiveness and ultimately the likelihood of toxicity. A review of the role of functional genomics on both physiological and evolutionary timescales illustrates the interaction between genetic variability and transcriptional response variability (Reid & Whitehead, 2016). In populations of *F. heteroclitus* adapted to contaminated environments, the transcriptional profiles of control individuals are rather indistinguishable from references (Fisher & Oleksiak, 2007; Oleksiak, 2008). On the other hand, when presented with stressors, the responses of adapted populations diverge from those of references (Fisher & Oleksiak, 2007; Oleksiak, 2008; Whitehead et al., 2010). This suggests not only that there is variability in the expression of genes in response to contaminants, which is encoded genetically, but that induced expression response profiles can be altered through natural selection in contaminated environments.

Functional genomics and transcriptomics are a fundamental combination, which when combined can elucidate the effects of selection on specific genotypes to alter population responses to contaminants. While population genomics can sometimes pinpoint the specific genotype responsible for adaptation, transcriptomics can begin to translate those changes into functional pathway alterations. Transcriptomics can reveal the networks that are perturbed by contaminants, while in a comparative context, it can reveal networks perturbed by evolutionary divergence. Thus, linking transcriptomic analysis to population genomics is a crucial next step in evolutionary toxicology that can relate genetic change to functional effects, a valuable step toward phenotypically anchoring genotypes.

The limitations of transcriptomics are twofold: standalone power of discrimination and understanding of the effects of altered gene expression on individual health. When transcriptomics is performed by itself, it can often be difficult to discern the meaning of highly variable profiles. For example, during development, gene expression is highly active and extremely difficult to interpret (Bozinovic, Sit, Hinton, & Oleksiak, 2011). Thus, to gain full understanding of the effects of a contaminant on gene expression, a full profile of responsiveness through multiple stages of development needs to be created (Bozinovic et al., 2011). In addition, transcriptomics can often be difficult to replicate and standardize between laboratories (Vidal‐Dorsch et al., 2016) and many transcriptomic responses to contaminants are highly variable in magnitude (Schirmer et al., 2010). Additionally, the level of their responsiveness is not always well translated into downstream effects or implications on organismal health. However, the activity and responsiveness of many pathways, like the AhR, are highly conserved in vertebrates and can be linked to survival (Hahn, 2002; Meyer et al., 2002). As our understanding of relevant molecular pathways improves, the importance of transcriptomics in evolutionary toxicology will certainly increase.

Another area of utility for transcriptomic studies in evolutionary toxicology stems from the need to better understand the full impacts of rapid evolution on the health of populations. When adaptation to contamination is identified, possible fitness costs are some of the first targets to assess threats to adapted populations (Clark & Di Giulio, 2012; Oziolor, Dubansky, Burggren, & Matson, 2016b). These investigations take a targeted approach to explore effects on pathways likely affected by adaptation (Clark & Di Giulio, 2012; Oziolor et al., 2016b). On the other hand, with top‐down ‐omics approaches, understanding of transcriptomic, metabolomic, and proteomic differences in responsiveness and basal levels in adapted populations may inform possible fitness costs in a much more thorough manner (Bahamonde, Feswick, Isaacs, Munkittrick, & Martyniuk, 2016; Bundy, Davey, & Viant, 2008; Monsinjon & Knigge, 2007). One example is a comparative transcriptomic approach that finds altered responsiveness in biocide resistant *Salmonella enterica* Typhimurium that further explain increased sensitivity of this strain to other antimicrobials (Curiao et al., 2016). Thus, the other current ‐omic methods can act as a complement to population genomics to further characterize a resistant phenotype and its effects on a population.

## 7 GENE EXPRESSION: PHENOTYPIC PLASTICITY OR TRANSITORY PHYSIOLOGICAL RESPONSE?

While transcriptomics can be a useful tool for understanding the response of an organism to contamination, there are several integral characteristics of a physiological response with regards to gene expression: basal expression, inducibility, and duration of response. Here we will use as an example the AhR pathway. It is established that in response to dioxin‐like contaminants (DLCs), the AhR pathway is activated, leading to the induction of downstream regulated genes (ex. CYP1A; Hahn, 2002). It is also known that both basal and maximal induction levels of protein activity among reference populations of *F. grandis* are very similar (Oziolor et al., 2016b). Additionally, anthropogenic evolution (evolution in response to anthropogenic stressors) has been shown to result in the down‐regulation of both basal and maximum induction levels for this pathway (Oziolor et al., 2014). This recalcitrant AhR pathway in adapted fish populations is regarded as a resistant phenotype.

Phenotypic plasticity has been defined as any ability of a genotype to produce different phenotypes, including short‐term alterations in gene expression (Fusco & Minelli, 2010). In toxicology on the other hand, the ability of enzyme systems to be induced in response to contaminants or other environmental cues simply represent a transitory physiological response, not a phenotype. Here we argue that the phenotype is better described based on basal expression or activity, inducibility, and timing of the response to stimuli, rather than the snapshot gene expression profile determined during a transitory physiological response. Thus, a change in an individual's ability to induce a toxicological response to a certain magnitude, or a change in the basal levels of a pathway due to previous exposure would be considered an alternate phenotype, and thus demonstration of phenotypic plasticity. Furthermore, if alterations persist trans‐generationally, this could be evidence for either epigenetic alteration or genetic adaptation to the original stressor that altered the basic phenotype (baseline, maximal levels, or duration). If this change persists through multiple generations, this can and has been used as evidence of evolution (Nacci, Champlin, Coiro, McKinney, & Jayaraman, 2002). We suggest that in the context of gene expression, phenotypic plasticity be used only to describe changes in basal expression, inducibility, or the duration of response due to gene‐environmental interactions.

## 8 APPLICATIONS OF EVOLUTIONARY TOXICOLOGY

Adaptation to contamination in local wild populations is a fascinating process, which leaves organisms capable of dealing with levels of contamination beyond the capacity of reference populations of the same species. These local adaptations have been termed “evolutionary rescue” (Heino & Hanski, 2001). The use of this terminology stressed the resiliency of genetic variation in natural populations and is often linked to an ability to withstand the increasing threats of climate change and other anthropogenic contributors (Bell, 2013; Bell & Gonzalez, 2009, 2011; Schiffers, Bourne, Lavergne, Thuiller, & Travis, 2013; Tallmon, Luikart, & Waples, 2004). In addition, this process has been more reliable when organisms are faced with gradual environmental deterioration, rather than a quick onset of a strong selective pressure (Hansen, Olivieri, Waller, Nielsen, & Ge, 2012; Lindsey, Gallie, Taylor, & Kerr, 2013).

Despite the utility of the concept of evolutionary rescue, when applied to toxicology, this idea may lead to a false sense of positivity toward the process of adapting to contamination. When discussing the implementations of our understanding of evolutionary toxicology in an applicable framework, such as environmental regulation, there should be no confusion as to the interpretation of evolutionary outcomes. Adaptation is a process that functions through reductions in the frequencies of less fit phenotypes, and the associated genotypes, through strong negative effects on reproduction or survival of sensitive individuals in exposed populations over multiple generations. In other words, significant mortality and/or reproductive effects are required for population adaptation or “evolutionary rescue,” which is why this process should not be considered positively from a regulatory standpoint, but rather as evidence of chronic increased mortality. Another issue of concern following rapid adaption is the potential for fitness costs, both mechanistically related to the adapted phenotype, or completely independent (Clark & Di Giulio, 2012; Harbeitner et al., 2013; Meyer & Di Giulio, 2003; Oziolor et al., 2016b). While fitness costs in adapted populations are of concern where evolutionary rescue has occurred, it is also imperative to understand if other species in the same contaminated environments have also been affected.

The fact that a few species have been shown to have adapted to contaminated environments does not preclude the likelihood that many species in the same environments have been negatively impacted. This could be reflected in altered levels of species richness, as well as the genetic diversity of nonadapted species in contaminated environments. Most studies focus on understanding adapted populations and species, but an understanding of the overall ecological health of the communities in an environment may serve as a better indicator of the degraded ecosystem. This process is difficult as many species may be migratory, in small numbers or difficult to sample. This is another case in which eDNA can be useful in evolutionary toxicology. With the increased ability to sample under‐represented species in environments, evolutionary toxicology can expand its understanding of ecosystems and community health through greater understanding of gene flow and genetic diversity in these systems. This will allow investigations to directly link environmental contamination to a change in genomic end points for a broader range of species and further understand the impacts of chronic exposure to heavily contaminated environments.

As evolution to contamination results from generations of elevated mortality or reproductive effects, there are current efforts to relate evolutionary toxicology studies to regulatory limits in aquatic environments (De Coninck, Janssen, & De Schamphelaere, 2014; Oziolor et al., 2016a). Currently, regulatory limits are driven by standard toxicological end points, such as mortality and changes in reproduction (Long, Field, & MacDonald, 1998; Long & MacDonald, 1998; MacDonald, Ingersoll, & Berger, 2000). On the other hand, considering that adaptation to contaminated environments is a result of such changes occurring over multiple generations, it seems that evolution can inform regulation and risk analysis. Meta‐analyses use studies in which population adaptation has occurred and compare the contamination levels measured in these environments to regulatory benchmarks (De Coninck et al., 2014; Oziolor et al., 2016a). These studies are complicated by the use of different numbers and mixtures of chemical congeners between evolutionary studies and those used to set regulatory limits (Oziolor et al., 2016a). Even with this limitation, researchers have documented evolutionary adaptation in environments with contamination levels below relevant regulatory benchmarks (Oziolor et al., 2016a). Such conclusions open the discussion for a more thorough discourse between evolutionary toxicology and environmental regulation. In this context, evolutionary toxicology studies should move to increase the comparability of their environmental chemistry data with the methods used for regulatory processes. By making these investigations more easily comparable, this field could move a step closer toward informing regulatory bodies and being applied in risk assessments and for environmental management.

## 9 CONCLUDING REMARKS

The field of evolutionary toxicology has made immense progress over the last few decades and has already begun to take advantage of the increased accessibility of ‐omic resources. Genomics improves our ability to understand the mechanisms of adaptation and the full effects of evolutionary rescue on a population genomic level. Transcriptomics, proteomics, and metabolomics allow us to better understand phenotypic linkages to genotypes, and to better understand potential population impacts. The future of evolutionary toxicology should bring this field closer to informing regulation and application to practical risk assessment, but this will require an effort to increase comparability. An additional component of the expanding utility of evolutionary toxicology is the expansion of potential study organisms through eDNA utilization. This promising method will allow for the analysis of population‐wide effects on less represented, endangered and/or difficult to collect species. In summary, evolutionary toxicology is a versatile field that will continue to advance by taking advantage of new technologies for improving our understanding of the transgenerational impacts of contaminants on populations.
